# Methyl 6-eth­oxy-3-phenyl-3a,4-dihydro-3*H*-chromeno[4,3-*c*]isoxazole-3a-car­boxylate

**DOI:** 10.1107/S1600536812051720

**Published:** 2013-01-04

**Authors:** G. Suresh, J. Srinivasan, M. Bakthadoss, S. Aravindhan

**Affiliations:** a Department of Physics, Presidency College (Autonomous), Chennai 600 005, India; b Department of Organic Chemistry, University of Madras, Chennai 600 025, India

## Abstract

In the title compound, C_20_H_19_NO_5_, the dihedral angle between the mean plane of the pyran ring (which has a half-chair conformation) and the benzene ring of the chromeno ring system is 7.21 (7)°. The dihedral angle between the mean plane of the chromeno ring system and the isoxazole ring is 21.78 (6)°, while the isoxazole ring forms a dihedral angle of 72.60 (8)° with the attached phenyl ring. In the crystal, mol­ecules are linked *via* pairs of C—H⋯O hydrogen bonds, forming inversion dimers with an *R* ^2^_2_(10) ring motif. These dimers are linked *via* C—H⋯N hydrogen bonds, forming chains along [001].

## Related literature

For the biological activity of chromenopyrroles, see: Caine (1993 ▶), and of benzopyran and isoxazolidine derivatives, see: Lin *et al.* (1996 ▶); Hu *et al.* (2004 ▶). For uses of isoxazole derivatives, see: Baraldi *et al.* (1987 ▶); Eddington *et al.* (2002 ▶). For related structures, see: Gangadharan *et al.* (2011 ▶); Swaminathan *et al.* (2011 ▶). For hydrogen-bond motifs, see: Bernstein *et al.* (1995 ▶).
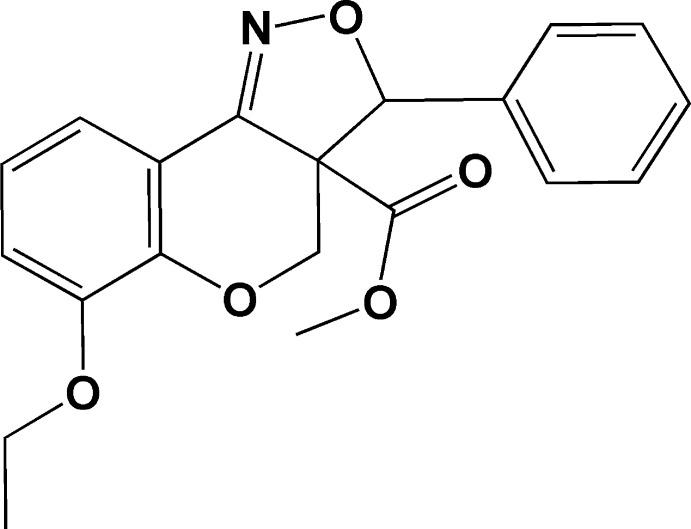


## Experimental

### 

#### Crystal data

C_20_H_19_NO_5_
*M* *_r_* = 353.36
Monoclinic, 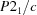

*a* = 12.9342 (6) Å
*b* = 7.5591 (2) Å
*c* = 18.7138 (8) Å
β = 105.440 (2)°
*V* = 1763.63 (12) Å^3^
*Z* = 4
Mo *K*α radiation
μ = 0.10 mm^−1^
*T* = 298 K
0.25 × 0.20 × 0.10 mm

#### Data collection

Bruker APEXII CCD area-detector diffractometer
Absorption correction: multi-scan (*SADABS*; Bruker, 2008 ▶) *T*_min_ = 0.976, *T*_max_ = 0.990
13064 measured reflections
4245 independent reflections
3253 reflections with *I* > 2σ(*I*)
*R*_int_ = 0.017

#### Refinement

*R*[*F* ^2^ > 2σ(*F* ^2^)] = 0.042
*wR*(*F* ^2^) = 0.125
*S* = 1.03
4245 reflections
237 parameters
H-atom parameters constrained
Δρ_max_ = 0.35 e Å^−3^
Δρ_min_ = −0.22 e Å^−3^

### 

Data collection: *APEX2* (Bruker, 2008 ▶); cell refinement: *SAINT* (Bruker, 2008 ▶); data reduction: *SAINT*; program(s) used to solve structure: *SHELXS97* (Sheldrick, 2008 ▶); program(s) used to refine structure: *SHELXL97* (Sheldrick, 2008 ▶); molecular graphics: *ORTEP-3* (Farrugia, 2012 ▶); software used to prepare material for publication: *SHELXL97* and *PLATON* (Spek, 2009 ▶).

## Supplementary Material

Crystal structure: contains datablock(s) I, global. DOI: 10.1107/S1600536812051720/su2543sup1.cif

Structure factors: contains datablock(s) I. DOI: 10.1107/S1600536812051720/su2543Isup2.hkl

Supplementary material file. DOI: 10.1107/S1600536812051720/su2543Isup3.cml

Additional supplementary materials:  crystallographic information; 3D view; checkCIF report

## Figures and Tables

**Table 1:** Hydrogen-bond geometry (Å, °)

*D*—H⋯*A*	*D*—H	H⋯*A*	*D*⋯*A*	*D*—H⋯*A*
C6—H6*A*⋯O4^i^	0.97	2.55	3.3875 (19)	145
C8—H8⋯N1^ii^	0.98	2.52	3.4269 (19)	154

Symmetry codes: (i) 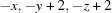; (ii) 
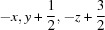
.

**Fig. 1.**
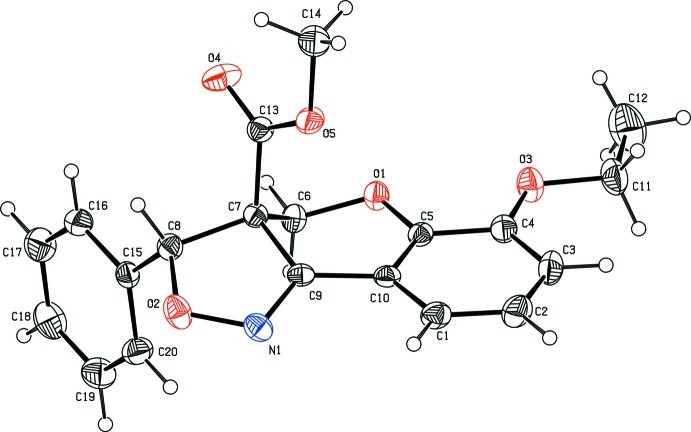
The molecular structure of the title molecule, with the atom numbering. Displacement ellipsoids are drawn at the 30% probability level.

**Fig. 2.**
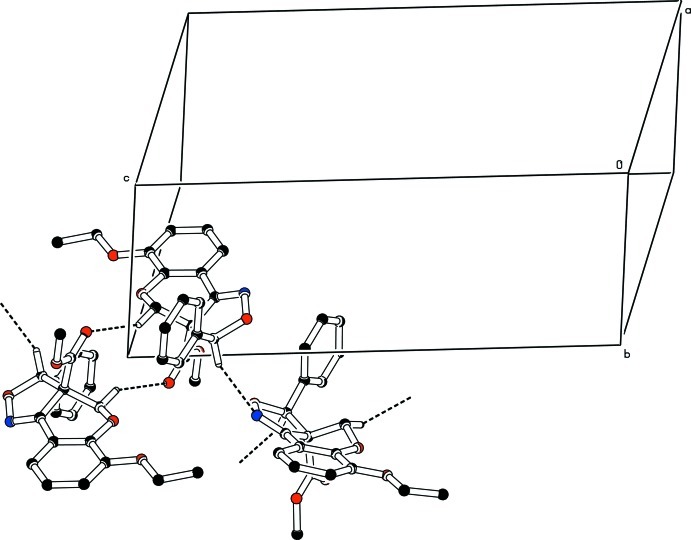
A partial view of the crystal packing of the title compound. The C-H···O and C-H···N hydrogen bonds are shown as dashed lines (see Table 1 for details).

## supplementary crystallographic information

### Comment

Chromenopyrrole compounds are used in the treatment of impulsive disorders (Caine, 1993). It is well known that benzo-pyran and isoxazolidine derivatives possess interesting biological and pharmacological activities (Lin *et al.*, 1996; Hu *et al.*, 2004). Isoxazole and its derivatives are key intermediates for the preparation of products which mimic natural compounds (Baraldi *et al.*, 1987). They have been shown to possess anticonvulsant activity (Eddington *et al.*, 2002). Herein we report on the synthesis and the crystal structure of the new title chromeno compound.

The molecular structure of the title molecule is illustrated in Fig. 1. In the chromeno ring system the pyran ring has a half chair conformation. Its mean plane makes a dihedral angle with the benzene ring of 7.21 (7)°. The dihedral angle between the mean plane of the chromeno ring system (fusion of benzene and pyran rings) and the isoxazole ring (O2/N1/C7-C9) is 21.78 (6)°. The isoxazole ring also forms a dihedral angle of 72.60 (8)° with the phenyl ring (C15—C20). The geometric parameters of the title molecule agree well with those reported for closely related structures (Gangadharan *et al.*, 2011; Swaminathan *et al.*, 2011).

In the crystal, molecules are linked via pairs of C-H···O hydrogen bonds to form inversion dimers with an *R*^2^_2_(10) ring motif (Bernstein *et al.*, 1995; Table 1 and Fig. 2). These dimers are linked via C-H···N hydrogen bonds to form chains along the c axis.

### Experimental

At 283 - 293 K, NCS (4 mmol) was added pinch wise over 3 h to a solution of ((*E*)-methyl2-((2ethoxy-6-((*E*)-(hydroxyimino)methyl)phenoxy)methyl) -3-phenylacrylate (2 mmol) in CCl_4_. After Et_3_N (4 mmol) was added to the reaction mixture which was stirred at room temperature for 2 h. After completion of the reaction, the mixture was evaporated under reduced pressure and the resulting crude mass was diluted with water (15 ml) and extracted with ethyl acetate (3 × 15 ml). The combined organic layers were washed with brine (2 × 10 ml) and dried over anhydrous Na_2_SO_4_. The organic layer was evaporated and purified by column chromatography (silica gel 60–120 mesh 7% EtOAc in hexanes) to provide the desired pure title product as a colourless solid. Crystals suitable for X-ray diffraction analysis were obtained by slow evaporation of a solution in ethyl acetate.

### Refinement

All the H atoms were placed in calculated positions and treated as riding atoms: C—H = 0.93–0.98 Å with *U*_iso_(H) = 1.5U_eq_(C) for methyl H atoms and = 1.2U_eq_(C) for other H atoms.

### Crystal data

**Table:**

C_20_H_19_NO_5_	*F*(000) = 744
*M**_r_* = 353.36	*D*_x_ = 1.331 Mg m^−^^3^
Monoclinic, *P*2_1_/*c*	Mo *K*α radiation, λ = 0.71073 Å
Hall symbol: -P 2ybc	Cell parameters from 4245 reflections
*a* = 12.9342 (6) Å	θ = 2.3–28.4°
*b* = 7.5591 (2) Å	µ = 0.10 mm^−^^1^
*c* = 18.7138 (8) Å	*T* = 298 K
β = 105.440 (2)°	Monoclinic, colourless
*V* = 1763.63 (12) Å^3^	0.25 × 0.20 × 0.10 mm
*Z* = 4	

### Data collection

**Table:**

Bruker APEXII CCD area-detector diffractometer	4245 independent reflections
Radiation source: fine-focus sealed tube	3253 reflections with *I* > 2σ(*I*)
Graphite monochromator	*R*_int_ = 0.017
ω and φ scans	θ_max_ = 28.4°, θ_min_ = 2.3°
Absorption correction: multi-scan (*SADABS*; Bruker, 2008)	*h* = −13→17
*T*_min_ = 0.976, *T*_max_ = 0.990	*k* = −9→6
13064 measured reflections	*l* = −24→25

### Refinement

**Table:**

Refinement on *F*^2^	Primary atom site location: structure-invariant direct methods
Least-squares matrix: full	Secondary atom site location: difference Fourier map
*R*[*F*^2^ > 2σ(*F*^2^)] = 0.042	Hydrogen site location: inferred from neighbouring sites
*wR*(*F*^2^) = 0.125	H-atom parameters constrained
*S* = 1.03	*w* = 1/[σ^2^(*F*_o_^2^) + (0.0578*P*)^2^ + 0.5069*P*] where *P* = (*F*_o_^2^ + 2*F*_c_^2^)/3
4245 reflections	(Δ/σ)_max_ = 0.002
237 parameters	Δρ_max_ = 0.35 e Å^−^^3^
0 restraints	Δρ_min_ = −0.22 e Å^−^^3^

### Special details

**Table:**

Geometry. All e.s.d.'s (except the e.s.d. in the dihedral angle between two l.s. planes) are estimated using the full covariance matrix. The cell e.s.d.'s are taken into account individually in the estimation of e.s.d.'s in distances, angles and torsion angles; correlations between e.s.d.'s in cell parameters are only used when they are defined by crystal symmetry. An approximate (isotropic) treatment of cell e.s.d.'s is used for estimating e.s.d.'s involving l.s. planes.
Refinement. Refinement of *F*^2^ against ALL reflections. The weighted *R*-factor *wR* and goodness of fit *S* are based on *F*^2^, conventional *R*-factors *R* are based on *F*, with *F* set to zero for negative *F*^2^. The threshold expression of *F*^2^ > σ(*F*^2^) is used only for calculating *R*-factors(gt) *etc*. and is not relevant to the choice of reflections for refinement. *R*-factors based on *F*^2^ are statistically about twice as large as those based on *F*, and *R*- factors based on ALL data will be even larger.

### Fractional atomic coordinates and isotropic or equivalent isotropic displacement parameters (Å^2^)

**Table:**

	*x*	*y*	*z*	*U*_iso_*/*U*_eq_	
C1	0.34469 (12)	0.7365 (2)	0.84801 (8)	0.0408 (3)	
H1	0.3280	0.7202	0.7969	0.049*	
C2	0.44803 (13)	0.7149 (2)	0.89052 (9)	0.0497 (4)	
H2	0.5013	0.6828	0.8680	0.060*	
C3	0.47417 (12)	0.7405 (2)	0.96675 (9)	0.0467 (4)	
H3	0.5449	0.7270	0.9945	0.056*	
C4	0.39633 (11)	0.78572 (19)	1.00188 (8)	0.0365 (3)	
C5	0.28961 (10)	0.80410 (17)	0.95890 (7)	0.0299 (3)	
C6	0.10418 (10)	0.81790 (19)	0.95728 (7)	0.0331 (3)	
H6A	0.0582	0.8683	0.9853	0.040*	
H6B	0.0895	0.6921	0.9516	0.040*	
C7	0.08137 (10)	0.90610 (17)	0.88085 (7)	0.0290 (3)	
C8	−0.02921 (11)	0.87517 (19)	0.82484 (7)	0.0341 (3)	
H8	−0.0613	0.9908	0.8088	0.041*	
C9	0.15362 (10)	0.81458 (16)	0.84177 (7)	0.0291 (3)	
C10	0.26470 (11)	0.78305 (17)	0.88215 (7)	0.0308 (3)	
C11	0.51955 (13)	0.7982 (3)	1.12204 (9)	0.0539 (4)	
H11A	0.5661	0.8839	1.1076	0.065*	
H11B	0.5473	0.6806	1.1177	0.065*	
C12	0.51486 (19)	0.8317 (4)	1.20010 (11)	0.0856 (7)	
H12A	0.4827	0.9451	1.2029	0.128*	
H12B	0.5862	0.8302	1.2326	0.128*	
H12C	0.4727	0.7413	1.2149	0.128*	
C13	0.10049 (11)	1.10449 (19)	0.89314 (7)	0.0338 (3)	
C14	0.20504 (14)	1.3490 (2)	0.87993 (12)	0.0608 (5)	
H14A	0.1388	1.4113	0.8606	0.091*	
H14B	0.2550	1.3815	0.8524	0.091*	
H14C	0.2342	1.3792	0.9312	0.091*	
C15	−0.10961 (10)	0.76672 (18)	0.85102 (7)	0.0331 (3)	
C16	−0.18456 (12)	0.8534 (2)	0.87901 (9)	0.0437 (4)	
H16	−0.1854	0.9764	0.8802	0.052*	
C17	−0.25822 (14)	0.7588 (3)	0.90523 (10)	0.0565 (4)	
H17	−0.3079	0.8180	0.9244	0.068*	
C18	−0.25809 (14)	0.5775 (3)	0.90297 (10)	0.0565 (4)	
H18	−0.3081	0.5139	0.9202	0.068*	
C19	−0.18410 (14)	0.4897 (2)	0.87527 (10)	0.0550 (4)	
H19	−0.1840	0.3667	0.8740	0.066*	
C20	−0.10973 (13)	0.5836 (2)	0.84930 (9)	0.0452 (4)	
H20	−0.0598	0.5236	0.8307	0.054*	
N1	0.10693 (10)	0.75033 (17)	0.77828 (6)	0.0376 (3)	
O1	0.21512 (7)	0.84634 (13)	0.99612 (5)	0.0351 (2)	
O2	−0.00309 (9)	0.79042 (16)	0.76155 (5)	0.0456 (3)	
O3	0.41260 (8)	0.81419 (16)	1.07581 (6)	0.0473 (3)	
O4	0.04263 (10)	1.19594 (15)	0.91752 (7)	0.0560 (3)	
O5	0.18555 (8)	1.16068 (13)	0.87330 (6)	0.0442 (3)	

### Atomic displacement parameters (Å^2^)

**Table:**

	*U*^11^	*U*^22^	*U*^33^	*U*^12^	*U*^13^	*U*^23^
C1	0.0428 (8)	0.0401 (8)	0.0440 (7)	0.0001 (6)	0.0196 (6)	−0.0057 (6)
C2	0.0400 (8)	0.0554 (10)	0.0602 (10)	0.0050 (7)	0.0244 (7)	−0.0042 (8)
C3	0.0295 (7)	0.0533 (10)	0.0561 (9)	0.0037 (6)	0.0093 (6)	0.0042 (7)
C4	0.0342 (7)	0.0344 (8)	0.0398 (7)	−0.0005 (5)	0.0081 (6)	0.0035 (6)
C5	0.0305 (6)	0.0247 (6)	0.0359 (6)	0.0000 (5)	0.0110 (5)	0.0013 (5)
C6	0.0304 (6)	0.0377 (7)	0.0326 (6)	0.0001 (5)	0.0106 (5)	0.0037 (5)
C7	0.0292 (6)	0.0276 (7)	0.0306 (6)	−0.0009 (5)	0.0087 (5)	0.0015 (5)
C8	0.0335 (7)	0.0340 (7)	0.0331 (6)	−0.0002 (5)	0.0057 (5)	0.0031 (5)
C9	0.0353 (7)	0.0228 (6)	0.0312 (6)	−0.0029 (5)	0.0125 (5)	0.0008 (5)
C10	0.0343 (7)	0.0228 (6)	0.0368 (6)	−0.0021 (5)	0.0121 (5)	−0.0019 (5)
C11	0.0421 (9)	0.0584 (11)	0.0511 (9)	−0.0016 (7)	−0.0051 (7)	0.0071 (8)
C12	0.0797 (15)	0.118 (2)	0.0469 (10)	−0.0033 (14)	−0.0051 (10)	0.0057 (12)
C13	0.0363 (7)	0.0312 (7)	0.0323 (6)	0.0019 (5)	0.0062 (5)	−0.0006 (5)
C14	0.0486 (10)	0.0267 (8)	0.0982 (14)	−0.0065 (7)	0.0038 (9)	0.0070 (8)
C15	0.0287 (6)	0.0345 (7)	0.0333 (6)	−0.0011 (5)	0.0033 (5)	0.0003 (5)
C16	0.0376 (8)	0.0377 (8)	0.0557 (9)	−0.0017 (6)	0.0124 (7)	−0.0071 (7)
C17	0.0406 (9)	0.0671 (12)	0.0677 (11)	−0.0065 (8)	0.0247 (8)	−0.0119 (9)
C18	0.0463 (9)	0.0633 (12)	0.0616 (10)	−0.0185 (8)	0.0177 (8)	0.0046 (9)
C19	0.0575 (10)	0.0367 (9)	0.0691 (11)	−0.0102 (7)	0.0139 (9)	0.0050 (8)
C20	0.0449 (8)	0.0344 (8)	0.0581 (9)	0.0015 (6)	0.0168 (7)	−0.0015 (7)
N1	0.0399 (7)	0.0405 (7)	0.0332 (5)	−0.0065 (5)	0.0112 (5)	−0.0032 (5)
O1	0.0313 (5)	0.0444 (6)	0.0297 (4)	0.0024 (4)	0.0082 (4)	−0.0010 (4)
O2	0.0386 (5)	0.0654 (7)	0.0316 (5)	−0.0081 (5)	0.0073 (4)	−0.0052 (5)
O3	0.0377 (6)	0.0623 (7)	0.0381 (5)	0.0015 (5)	0.0036 (4)	0.0023 (5)
O4	0.0714 (8)	0.0398 (6)	0.0659 (7)	0.0076 (6)	0.0343 (6)	−0.0079 (5)
O5	0.0364 (5)	0.0245 (5)	0.0706 (7)	−0.0013 (4)	0.0123 (5)	0.0041 (4)

### Geometric parameters (Å, º)

**Table:**

C1—C2	1.371 (2)	C11—C12	1.500 (3)
C1—C10	1.3980 (19)	C11—H11A	0.9700
C1—H1	0.9300	C11—H11B	0.9700
C2—C3	1.389 (2)	C12—H12A	0.9600
C2—H2	0.9300	C12—H12B	0.9600
C3—C4	1.384 (2)	C12—H12C	0.9600
C3—H3	0.9300	C13—O4	1.1949 (17)
C4—O3	1.3600 (17)	C13—O5	1.3215 (17)
C4—C5	1.4073 (18)	C14—O5	1.4451 (19)
C5—O1	1.3685 (16)	C14—H14A	0.9600
C5—C10	1.3948 (18)	C14—H14B	0.9600
C6—O1	1.4416 (16)	C14—H14C	0.9600
C6—C7	1.5337 (17)	C15—C16	1.383 (2)
C6—H6A	0.9700	C15—C20	1.385 (2)
C6—H6B	0.9700	C16—C17	1.382 (2)
C7—C9	1.5003 (18)	C16—H16	0.9300
C7—C13	1.5273 (19)	C17—C18	1.371 (3)
C7—C8	1.5494 (17)	C17—H17	0.9300
C8—O2	1.4630 (17)	C18—C19	1.375 (3)
C8—C15	1.5049 (19)	C18—H18	0.9300
C8—H8	0.9800	C19—C20	1.384 (2)
C9—N1	1.2767 (17)	C19—H19	0.9300
C9—C10	1.4540 (18)	C20—H20	0.9300
C11—O3	1.4281 (18)	N1—O2	1.4063 (16)
			
C2—C1—C10	119.37 (14)	O3—C11—H11B	110.3
C2—C1—H1	120.3	C12—C11—H11B	110.3
C10—C1—H1	120.3	H11A—C11—H11B	108.6
C1—C2—C3	120.84 (14)	C11—C12—H12A	109.5
C1—C2—H2	119.6	C11—C12—H12B	109.5
C3—C2—H2	119.6	H12A—C12—H12B	109.5
C4—C3—C2	120.88 (14)	C11—C12—H12C	109.5
C4—C3—H3	119.6	H12A—C12—H12C	109.5
C2—C3—H3	119.6	H12B—C12—H12C	109.5
O3—C4—C3	125.96 (13)	O4—C13—O5	125.05 (14)
O3—C4—C5	115.37 (12)	O4—C13—C7	122.19 (13)
C3—C4—C5	118.67 (13)	O5—C13—C7	112.76 (11)
O1—C5—C10	123.36 (12)	O5—C14—H14A	109.5
O1—C5—C4	116.64 (11)	O5—C14—H14B	109.5
C10—C5—C4	119.98 (12)	H14A—C14—H14B	109.5
O1—C6—C7	108.90 (10)	O5—C14—H14C	109.5
O1—C6—H6A	109.9	H14A—C14—H14C	109.5
C7—C6—H6A	109.9	H14B—C14—H14C	109.5
O1—C6—H6B	109.9	C16—C15—C20	119.08 (14)
C7—C6—H6B	109.9	C16—C15—C8	118.66 (13)
H6A—C6—H6B	108.3	C20—C15—C8	122.25 (13)
C9—C7—C13	115.42 (11)	C17—C16—C15	120.52 (15)
C9—C7—C6	105.31 (10)	C17—C16—H16	119.7
C13—C7—C6	107.65 (11)	C15—C16—H16	119.7
C9—C7—C8	100.56 (10)	C18—C17—C16	120.00 (16)
C13—C7—C8	109.47 (10)	C18—C17—H17	120.0
C6—C7—C8	118.62 (11)	C16—C17—H17	120.0
O2—C8—C15	110.70 (11)	C17—C18—C19	120.09 (16)
O2—C8—C7	104.08 (10)	C17—C18—H18	120.0
C15—C8—C7	117.10 (10)	C19—C18—H18	120.0
O2—C8—H8	108.2	C18—C19—C20	120.22 (16)
C15—C8—H8	108.2	C18—C19—H19	119.9
C7—C8—H8	108.2	C20—C19—H19	119.9
N1—C9—C10	125.34 (12)	C19—C20—C15	120.09 (15)
N1—C9—C7	115.30 (12)	C19—C20—H20	120.0
C10—C9—C7	118.67 (11)	C15—C20—H20	120.0
C5—C10—C1	120.21 (13)	C9—N1—O2	108.97 (11)
C5—C10—C9	116.22 (11)	C5—O1—C6	116.72 (10)
C1—C10—C9	123.56 (12)	N1—O2—C8	110.62 (9)
O3—C11—C12	106.98 (16)	C4—O3—C11	117.75 (12)
O3—C11—H11A	110.3	C13—O5—C14	115.58 (13)
C12—C11—H11A	110.3		
			
C10—C1—C2—C3	−0.6 (2)	C7—C9—C10—C1	−165.09 (13)
C1—C2—C3—C4	1.0 (3)	C9—C7—C13—O4	173.18 (13)
C2—C3—C4—O3	−179.88 (15)	C6—C7—C13—O4	−69.56 (16)
C2—C3—C4—C5	0.5 (2)	C8—C7—C13—O4	60.65 (17)
O3—C4—C5—O1	−0.61 (18)	C9—C7—C13—O5	−6.14 (16)
C3—C4—C5—O1	179.04 (13)	C6—C7—C13—O5	111.12 (12)
O3—C4—C5—C10	178.04 (12)	C8—C7—C13—O5	−118.67 (12)
C3—C4—C5—C10	−2.3 (2)	O2—C8—C15—C16	−146.00 (12)
O1—C6—C7—C9	61.42 (13)	C7—C8—C15—C16	94.96 (15)
O1—C6—C7—C13	−62.23 (13)	O2—C8—C15—C20	35.24 (17)
O1—C6—C7—C8	172.88 (11)	C7—C8—C15—C20	−83.80 (17)
C9—C7—C8—O2	−5.46 (12)	C20—C15—C16—C17	0.3 (2)
C13—C7—C8—O2	116.48 (12)	C8—C15—C16—C17	−178.49 (14)
C6—C7—C8—O2	−119.52 (12)	C15—C16—C17—C18	−0.6 (3)
C9—C7—C8—C15	117.06 (12)	C16—C17—C18—C19	0.6 (3)
C13—C7—C8—C15	−121.00 (13)	C17—C18—C19—C20	−0.2 (3)
C6—C7—C8—C15	3.00 (18)	C18—C19—C20—C15	−0.1 (2)
C13—C7—C9—N1	−115.20 (13)	C16—C15—C20—C19	0.0 (2)
C6—C7—C9—N1	126.23 (12)	C8—C15—C20—C19	178.80 (13)
C8—C7—C9—N1	2.45 (15)	C10—C9—N1—O2	172.22 (12)
C13—C7—C9—C10	73.79 (14)	C7—C9—N1—O2	1.90 (16)
C6—C7—C9—C10	−44.78 (15)	C10—C5—O1—C6	18.01 (17)
C8—C7—C9—C10	−168.57 (11)	C4—C5—O1—C6	−163.39 (12)
O1—C5—C10—C1	−178.77 (13)	C7—C6—O1—C5	−50.96 (15)
C4—C5—C10—C1	2.7 (2)	C9—N1—O2—C8	−5.87 (15)
O1—C5—C10—C9	2.05 (18)	C15—C8—O2—N1	−119.54 (12)
C4—C5—C10—C9	−176.51 (12)	C7—C8—O2—N1	7.10 (14)
C2—C1—C10—C5	−1.2 (2)	C3—C4—O3—C11	0.7 (2)
C2—C1—C10—C9	177.92 (14)	C5—C4—O3—C11	−179.68 (13)
N1—C9—C10—C5	−155.97 (13)	C12—C11—O3—C4	−178.77 (16)
C7—C9—C10—C5	14.06 (17)	O4—C13—O5—C14	−2.3 (2)
N1—C9—C10—C1	24.9 (2)	C7—C13—O5—C14	176.95 (12)

### Hydrogen-bond geometry (Å, º)

**Table:**

*D*—H···*A*	*D*—H	H···*A*	*D*···*A*	*D*—H···*A*
C6—H6*A*···O4^i^	0.97	2.55	3.3875 (19)	145
C8—H8···N1^ii^	0.98	2.52	3.4269 (19)	154

Symmetry codes: (i) −*x*, −*y*+2, −*z*+2; (ii) −*x*, *y*+1/2, −*z*+3/2.
